# Beyond the trial: a single-center retrospective review of real-world lupus nephritis management and outcomes

**DOI:** 10.3389/fmed.2026.1868000

**Published:** 2026-07-21

**Authors:** Dylan Clapp, Mugdha Kulkarni, Anthony C. Leonard, Manish Anand

**Affiliations:** 1 University of Cincinnati College of Medicine, Cincinnati, OH, United States; 2 Department of Nephrology, Geisinger College of Health Sciences, Scranton, PA, United States; 3 Department of Biostatistics, Health Informatics, and Data Sciences, University of Cincinnati College of Medicine, Cincinnati, OH, United States; 4 Department of Internal Medicine, Division of Nephrology, University of Cincinnati College of Medicine, Cincinnati, OH, United States

**Keywords:** glomerulonephritis, immunosuppression, lupus nephritis, renal outcomes, retrospective cohort, systemic lupus erythematosus

## Abstract

**Background:**

Systemic lupus erythematosus (SLE) is a prevalent autoimmune disease affecting up to a quarter million Americans. Lupus nephritis (LN) is an immune complex glomerulonephritis that occurs when circulating immune complexes deposit in the kidney and is a common sequela of SLE. LN serves as a marker of disease severity and contributes significantly to mortality in SLE. Despite growing access to novel therapies, the disease burden remains high, and the gap between real-life challenges and clinical trial success persists. Our study is an effort to bridge the gap by examining factors such as diverse choice of immunosuppression, socioeconomic constraints, and adherence outside the carefully calibrated environment of clinical trials.

**Methods:**

We conducted a retrospective review of patients with biopsy proven LN to evaluate prognostic factors and treatment related complications at a tertiary hospital with patients selected from biopsy records obtained from January 2015 through December 2023. A total of 66 patients met inclusion criteria and were included in the final analysis of the cohort. Patients with findings other than confirmed LN and kidney transplant recipients were excluded. Longitudinal data were collected at 6 months, 12 months, 2 years, and 5 years after biopsy, and response to treatment was defined using Kidney Disease Improving Global Outcomes (KDIGO) 2024 clinical practice guidelines for LN.

**Results:**

Of the 163 kidney biopsy reports reviewed from January 2015 through December 2023, 66 were confirmed as LN and included in the analysis. The median age at biopsy was 35 years, 76% were women, and 62% were African American. At 6 months, 50% of Class I and II LN, 49% of Class III, IV, III + V and IV + V LN, and 83% of Class V LN had no response to treatment, without significant differences between classes or in terms of partial vs. complete response. At 12 months, 55% of Class I and II, 50% of Class III, IV, III + V, and IV + V, and 87% of Class V LN had no response to treatment, with a significant difference (*p* = 0.04) between classes in terms of rate of response, but not in terms of partial vs. complete response. Notable complications were advancement to ESRD in 11 patients (17%), infections that required hospital admission in 27 patients (41%), and death in 9 patients (14%) in the study period.

**Conclusion:**

Our study provides practical and real-world data on management of LN. In patients with SLE, LN is associated with significant morbidity and mortality. Management continues to pose a unique challenge despite developing therapies. Sustained remission is often dependent on a complex interplay of individualized care, patient adherence, and socioeconomic factors. There is a need for increased awareness of LN as early biopsy, prompt initiation and continuation of treatment positively impacts outcomes.

## 1 Introduction

Systemic lupus erythematosus (SLE) is an autoimmune disease that affects almost 204,000 Americans, with a global prevalence estimated to range from 48–366.6 cases per 100,000 people (1, 2). The disease is caused by autoantibodies directed against cellular components that form circulating immune complexes, which then deposit in tissues throughout the body. The resulting inflammation produces the multisystem disease observed in SLE.

Lupus nephritis (LN) is an immune complex glomerulonephritis that occurs when circulating immune complex deposits in the kidney and is a common sequela of the disease (1). Up to 50% of patients with SLE develop LN. Women are more often affected than men, with 90% of LN patients being women. It often presents at younger ages, most commonly between 15–44 years and is more frequent among people of African American, Asian American, Hispanic, Native American, and Pacific Islander descent than in Caucasians (3).

LN is a key prognostic factor in the overall SLE disease process, with renal damage being the most important predictor of mortality (4). Despite treatment, 25% of all LN patients and around 50% of patients with Class IV LN develop end stage renal disease (ESRD) within 10 years of diagnosis (5, 6).

Diagnosis requires renal biopsy, which provides histopathological confirmation and classification of LN. The International Society of Nephrology/Renal Pathology Society (ISN/RPS) classification 2018 divides LN into six classes based on patterns of glomerular injury, which are important for both prognosis and therapeutic decision-making (7). Biopsy data allows for contextualization of disease activity and severity which guides approaches to treatment.

Prompt and aggressive immunosuppression is the holy grail of the LN treatment to halt renal damage. Treatment is typically divided into two stages: induction, where high-dose immunosuppression is administered in the initial months to induce remission, and maintenance, where lower-dose therapy is continued after disease remission to prevent disease relapses. Common induction agents include cyclophosphamide, mycophenolate, and corticosteroids, while maintenance therapy most often consists of mycophenolate or azathioprine, frequently in combination with low-dose steroids (4, 9). Despite newer therapeutic options, LN remains difficult to treat, with high relapse rates, progression to ESRD, and treatment-related complications such as infection, cardiovascular disease, avascular necrosis, osteoporosis, and malignancy (9). There is a growing repertoire of immunosuppressive options for LN, however, overall morbidity and mortality remains a challenge. Various studies have investigated biochemical markers and treatment options to prognosticate and treat the disease, however, outside the carefully calibrated trial environment, real world obstacles alter the course of the disease and affect outcomes.

Given these challenges, real-world data are essential to improve our understanding of outcomes and complications in LN. However, published reports are limited, and outcomes vary widely across centers and patient populations. A single-center review allows for consistent biopsy interpretation, uniform follow-up, and detailed treatment capture. The objective of this study was to describe the demographics, clinical features, treatment outcomes, and complications of patients with biopsy-proven LN treated at our institution over an eight-year period (2015–2023). This data can serve to bridge the gap between carefully controlled clinical trials and real-world clinical practice.

## 2 Methods

We conducted a retrospective review of patients with biopsy proven LN with a primary objective to evaluate treatment outcomes, prognostic factors, and complications experienced in true clinical practice at a tertiary hospital. Participants were selected from biopsy records during the period of January 2015 through December 2023. The biopsy library was searched with keywords “Lupus” and “Nephritis,” which yielded a total of 163 kidney biopsy reports. To be included, patients must have had an ISN/RPS histological classification of LN using the updated 2018 guidelines (7). Only patients with native kidney biopsies were included, all transplant biopsies were excluded. Reports that mentioned “not suggestive of lupus nephritis” or indicated the presence of other primary glomerular diseases were also excluded. Of the 163 biopsy reports obtained, 66 met criteria as confirmed LN were included in the study. Some patients underwent repeat biopsies during the study period however only the earliest biopsy was included for analysis purposes. No formal SLE diagnostic criteria were used as inclusion parameters as many patients already had a diagnosis of SLE and were established with rheumatology at the time of biopsy. If a patient did not have an SLE diagnosis at biopsy, histopathologic identification of LN was used as the marker of a new SLE diagnosis.

Histopathology data included the ISN/RPS 2018 classification of LN and, where available, along with activity and chronicity indices. Longitudinal data were collected at 6 months, 12 months, 2 years, and 5 years after biopsy, including eGFR (defined by CKD-EPI), serum creatinine, and urine protein: creatinine ratio (UPCR) calculated from spot urine sample. Due to the retrospective, observational nature of the study, exact follow up at these time points was not always available. The closest follow-up within 2 months of each of these follow up periods was used for the sample. If a patient did not have data for a given follow-up period, then they were not included in the analysis at that time point but could be included in later analyses if they returned for follow-up at a later date. In all tables and analyses, populations include only the patients that met criteria for that analysis. For Partial Response vs. Complete Response, only the patients that met criteria for any response were included in that analysis, so the denominator includes only the available patients in the partial and complete response groupings.

ESRD was defined as an eGFR less than 15 mL/min/1.73 m^2^ after initiation of treatment for LN irrespective of dialysis initiation. Acute kidney injury (AKI) was defined using KDIGO 2012 guidelines as any of the following: an increase in serum creatinine of more than 0.3 mg/dL within 48 h, or serum creatinine 1.5 times the patient's baseline known or presumed to have occurred in the previous 7 days, or urine output less than 0.5 mL/kg/h over the prior 6 h (8).

Using Kidney Disease Improving Global Outcomes (KDIGO) 2024 clinical practice guidelines for LN, response rates for patients with LN were determined at 6 months and 12 months. Complete response (CR) was defined as proteinuria less than 0.5 g/g with normal or stable serum creatinine (≤15% increase from baseline). Partial response (PR) was defined as ≥50% reduction in proteinuria to sub-nephrotic range with stable or improved creatinine (10). Any response was defined as either a partial or complete response, while no response was defined as neither. Primary efficacy renal response (PERR) occurred when UPCR is < 0.7 g/g and eGFR is stabilized to at least 60% of baseline (11).

Relapses (flare) were defined as recurrence or worsening of proteinuria or increase in creatinine meeting KDIGO criteria after achievement of PR or CR. This occurs when patients met criteria for achieving renal response, then later presented with proteinuria, hematuria, or kidney injury meeting criteria for biopsy or need for induction treatment (10).

Induction treatment was defined as the initial high dose immunosuppression given in the first 6 months of treatment. Maintenance treatment was the medication that was continued from 6 months forward, often at a reduced dose compared to induction.

Complications were classified into two categories: treatment-related complications and biopsy-related complications (see Supplementary File S1). Treatment-related complications included infections requiring admission, and malignancy following treatment of a patient's LN. Biopsy-related complications included bleeding and hematoma attributed to the procedure itself. Induction and maintenance regimens were recorded, and analysis of complications and responses between common induction regimens was completed.

De-identified data from the study cohort were used for statistical analysis. When appropriate, means with standard deviations (SD) or medians with interquartile ranges (IQR) were used to summarize continuous variables. Counts and percentages were used to summarize categorical variables. Because of the small sample size and distribution of categorical variables, two-tailed Fisher's exact tests were used to compare groups. The relationships between each of activity and chronicity scores and 12-month response rates were examined using logistic regressions. Missing data points were left missing, without use of data imputation. Statistical significance was assessed using a two-sided alpha threshold of 0.05, unadjusted for multiple tests. All analyses were conducted using SAS 9.4 statistical software.

## 3 Results

### 3.1 Baseline characteristics

Of the 163 kidney biopsy reports reviewed from January 2015 through December 2023, 66 were confirmed as LN and included in the analysis. The median age at biopsy was 35 years, 76% were women, and 62% were African American. Table 1 shows most baseline demographics of the study population.

**Table 1:** Demographics and baseline clinical characteristics.

Characteristics	Value
Age (yr.) at biopsy, median (IQR)	35 (25–49)
Sex *N* (%)	
*Female*	50 (76)
*Male*	16 (24)
Race *N* (%)	
*African American*	41 (62)
*Asian*	10 (15)
*White*	11 (17)
*American Indian/Alaskan Native*	1 (2)
*Hispanic*	2 (3)
*Multi-racial*	1 (2)
Duration (months) of SLE prior to biopsy, median (IQR)	12 (0–75)
Lupus nephritis classification *N* (%)	
*Class I*	1 (2)
*Class II*	10 (15)
*Class III*	10 (15)
*Class III, V*	10 (15)
*Class IV*	14 (21)
*Class IV, V*	6 (9)
*Class V*	15 (23)
Activity score, mean (SD)	6.4 (5.7)
Chronicity score, mean (SD)	2.8 (2.4)
RAAS^#^ Inhibitors prior to biopsy *N* (%)	35 (53)
Hydroxychloroquine *N* (%)	56 (85)
Serum creatinine mg/dL, mean (SD)	1.5 (1.3)
AKI at biopsy *N* (%)	18 (28)
*Classes I/II*	2/11 (18)
*Classes III/IV/III+V/IV+V*	8/40 (20)
*Class V*	8/14 (57)
eGFR ml/min/1.73 m^2^, mean (SD)	63.7 (30.8)
*Class I/II*	74.2 (28.7)
*Class III/IV/III+V/IV+V*	67.5 (30.3)
*Class V*	48 (29.9)
Serum albumin g/dL, mean (SD)	3.0 (0.7)
Systolic blood pressure mmHg, mean (SD)	131 (22)
Diastolic blood pressure mmHg, mean (SD)	77 (13)
C3 level mg/dL, mean (SD)	74.7 (31.3)
C4 level mg/dL, mean (SD)	16.8 (10.9)
ANA positive *N* (%)	57 (97)
Anti-DSDNA positive *N* (%)	54 (82)
Anti-phospholipid antibodies positive *N* (%) ^*^	37 (56)

* Defined as B2-glycoprotein, anticardiolipin, or lupus anticoagulant.

# Renin-Angiotensin-Aldosterone System Inhibitors.

46 of the 66 (70%) patients lacked secondary insurance or had no insurance. The most common LN classification was Class V (15 patients, 23%).

At the time of biopsy, the mean UPCR was 3.2 g/g, and the mean serum creatinine was 1.5 mg/dL. 28 (43%) patients had nephrotic range proteinuria (greater than 3 g/g) at the time of biopsy. The average activity and chronicity indices were available for 61 and 60 of the 66 patients with mean values of 6.4 and 2.8, respectively. The median time from SLE diagnosis to biopsy-proven LN was 12 months.

Many patients were receiving treatment for extrarenal SLE manifestations; 85% (56/66) were on hydroxychloroquine at the time of biopsy. Fifty-three percent (35/66) were on RAAS inhibitors prior to biopsy.

**Figure 1:**
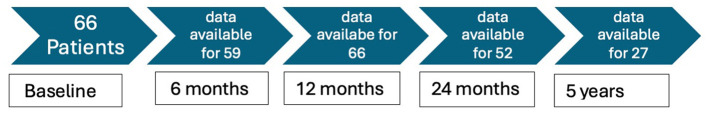
Patient flow diagram indicating available data at each of the longitudinal follow-up periods.

Figure 1 shows a patient flow diagram indicating patient follow-up and available data at each analyzed time point.

#### 3.1.1 Acute kidney injury

At the time of biopsy, 18 patients met clinical criteria for AKI. Among these patients, 2 had class I/II LN, 8 had proliferative LN (i.e., class III, class IV, with or without class V), and 8 were class V LN only. Rates of AKI significantly differed among classes (*p* = 0.03). The recovery rate for AKI among the classes was 100%, 50%, and 38% respectively (*p* = 0.54). Table 2 summarizes data on AKI.

**Table 2:** Acute kidney injury, ESRD progression, and death.

LN Class	N in Class	Number with AKI	AKI Recovered *N* (%)	ESRD *N* (%)	Death *N* (%)
1, 2	11	2/11	2/2 (100)	0/11 (0)	2/11 (18)
3, 4, 3 + 5, 4 + 5	40	8/40	4/8 (50)	6/39 (15)	6/40 (15)
5	15	8/14	3/8 (38)	5/13 (38)	1/14 (7)

Recovery rates include only the patients in each LN class group that had an AKI at biopsy. The rate of AKI differed significantly between classes, (*p* = 0.03), the rates of recovery from AKI did not (*p* = 0.54). Progression to ESRD differed significantly by class (*p* = 0.04). AKI data is missing for 1 patient. ESRD data is missing for 3 patients.

### 3.2 Induction regimens

**Figure 2:**
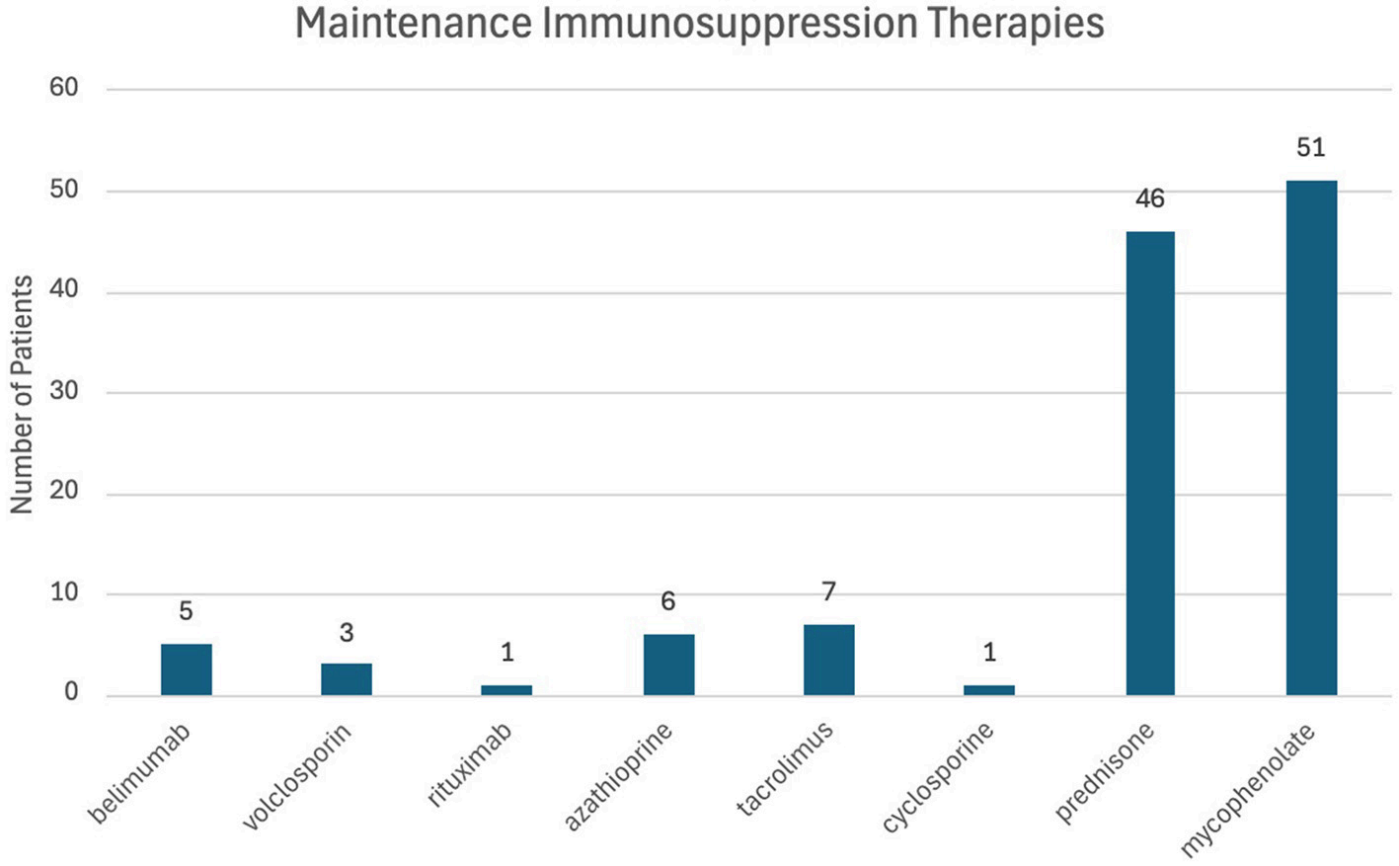
Maintenance immunosuppression. Numbers do not add to 66 as some patients were on multiple forms of maintenance therapy, i.e., mycophenolate and prednisone.

Patients were grouped into four induction categories: (1) Cyclophosphamide (CYC) with or without intravenous methylprednisolone (IVMP), (2) IVMP only, (3) Mycophenolate mofetil (MMF), with or without IVMP, and (4) No immunosuppression. MMF was the most used induction agent in 65% of patients. Induction selection varied significantly by LN class (*p* < 0.0001). Consistent with KDIGO guidelines, Class I and II disease most commonly did not receive induction therapy (64%) whereas patients with proliferative LN (Classes III, IV, III and V, IV and V) most often received MMF-based regimens. Of the 66 patients, 57 received oral glucocorticoids (86%) with a median dose of 50 mg daily. Induction regimens and maintenance medications are detailed in Table 3 and Figure 2.

**Table 3:** Selected induction regimens by LN classification.

Lupus Class	N in Class	Induction medications *N* (%)			
		Cyclophosphamide + IVMP	MMF +/- IVMP	IVMP alone	No Induction
1, 2	11	0 (0)	3 (75)	1 (25)	7 (10.6)
3, 4, 3+5, 4+5	40	10 (25)	28 (72)	1 (3)	1 (1.5)
5	15	1 (7)	12 (86)	1 (7)	1 (1.5)

There was significant difference between classes in terms of induction medication selected (*p* < 0.0001). Induction data is missing for 2 patients.

### 3.3 Renal response outcomes

Renal responses were assessed at 6 and 12 months, categorized as no response, PR, CR, and PERR. Seven patients lacked 6-month follow-up data; all had 12-month data available. At 6 months, 50% of Class I–II LN patients had no response, of the 4 that responded to treatment, 1 had PR, and 3 had CR. 49% patients with LN Classes III, IV, III + V, and IV + V LN had no response. In the proliferative LN group, total responders including complete and partial were 20. 11/20 had a PR whereas 9 had a CR.

In the Class V LN patients, 83% had no response at 6 months. 2 patients with Class V LN responded at 6 months, and both met criteria for CR. There was not a significant difference between classes in terms of response to therapy (*p* = 0.10), or in the rates of complete response groups (*p* = 0.34) at 6 months.

At 12 months, 55% of LN Classes I–II, 50% of Classes III/IV/mixed, and 87% of Class V patients did not respond to treatment. In LN Classes I-II, 1 patient had a PR at 12 months and 4 had CR. In proliferative LN, 20 patients responded to treatment at 12 months, 5 (25%) had PR, and 15 (75%) had CR. In Class V LN only 2 (13%) patients were responders at 12 months, 1 PR, and 1 with CR. At 12 months there was a significant difference between classes for response to therapy (*p* = 0.04). The rates of complete response between classes were not significant (*p* = 0.78). The response data are detailed in Table 4.

**Table 4:** Renal response data.

LN Class	*N* in Class	No response (6 mo.) *N* (%)	Partial response (6 mo.) *N* (%)	Complete response (6 mo.) *N* (%)	No response (12 mo.) *N* (%)	Partial response (12 mo.) *N* (%)	Complete response (12 mo.) *N* (%)
1, 2	11	4/8 (50)	1/4 (25)	3/4 (75)	6/11 (55)	1/5 (20)	4/5 (80)
3, 4, 3+5, 4+5	40	19/39 (49)	11/20 (55)	9/20 (45)	20/40 (50)	5/20 (25)	15/20 (75)
5	15	10/12 (83)	0/2(0)	2/2 (100)	13/15 (87)	1/2 (50)	1/2 (50)

For partial and complete response, only patients that met criteria for response were included in the percentage. At 6 months, 7 patients did not have response data and were not included in the response percentages, while at 12 months all patients were evaluated. Rates for partial and complete responses are for only those patients who had either type of response. Difference between partial and complete response were not significant at either 6 months or 12 months (*p* = 0.34, and *p* = 0.78 respectively). At 6 months there was no difference in rates of response vs. No Response (*p* = 0.10), at 12 months there was significant difference (*p* = 0.04).

Renal response at 12 months was evaluated based on activity score and chronicity score at biopsy with no relationship noted between rates of response and either activity score (*p* = 0.47) or chronicity score (*p* = 0.39).

Using the PERR definition of renal response at 6 months, 26 (39%) patients across all classes satisfied criteria for inclusion. At 12 months, 3 patients that met PERR criteria at 6 months had worsening of proteinuria and no longer qualified for PERR. However, an additional 12 patients across all classes met PERR criteria for a total of 35 (53%) patients attaining PERR.

At 2 years 52 patients had eGFR data available. 5 of 52 had CKD IV and another 5 of 52 patients developed ESRD. At 5 years, eGFR data was only available for 23 of the initial 66 patients, and 7 of 23 had progressed to advanced CKD with eGFR less than 30 mL/min.

### 3.4 Complications

#### 3.4.1 Infectious complications

Twenty-seven patients (41%) experienced infections requiring hospital admission. Patients receiving cyclophosphamide or MMF had similar infection rates (36% and 35%, respectively). All three patients treated with IVMP alone had infectious complications. In this cohort, there were 9 patients who were on oral steroids only and 56% (5/9) of those patients had infectious complications requiring hospitalization. Four patients had COVID-19 infection. Differences in infection rates across all four induction groups were not significant (*p* = 0.14). Data are summarized in Table 5. All infections are listed in the Supplementary File S1.

**Table 5:** Infections requiring hospital admission by selected induction regimens.

Induction medication	N in medication group	Number (%) with infections
Cyclophosphamide + IVMP	11	4/11 (36)
MMF +/- IVMP	43	15/43 (35)
IVMP Alone	3	3/3 (100)
No Induction	9	5/9 (56)

Rates of infection did not differ significantly across induction groups (*p* = 0.14).

#### 3.4.2 Non-infectious complications

Four patients had retroperitoneal hematomas following biopsy but none of them were organ threatening or life threatening. Two patients were noted to have malignancy, one had Burkitt's Lymphoma and other had Craniopharyngioma.

Most of the patients were on steroids. 10 patients from the study group had diabetes and 3 of them were diagnosed after initial biopsy.

#### 3.4.3 Progression to ESRD, mortality, and disease flares

Progression to ESRD occurred in 11 patients (17%). In patients with Class I and Class II LN, no patient progressed to ESRD during the period analyzed (0/11). Those patients with proliferative LN 15% (6/39) progressed to ESRD. Finally, 38% (5/13) of patients with Class V LN developed ESRD. Three patients were missing follow up data and were not included in the analysis of ESRD progression. Across classes, differences in the rate of ESRD progression were significant (*p* = 0.04). Any renal response was significantly associated with progression to ESRD (*p* = 0.02), with 1 of 27 (4%) patients with renal response progressing to ESRD, as opposed to 10 of 36 non-responders (28%). Nine patients (14%) died during the study period. Out of the 9 deaths, 4 patients had ESRD at the time of death. Cause of death per death note was sepsis in 2 patients, lymphoma with tumor lysis syndrome, subarachnoid hemorrhage, hypoxic respiratory failure, and cardiogenic shock in others. For 2 patients the cause of death could not be found by chart review. One patient died during sleep and an autopsy was not performed. All causes of death are listed in the Supplementary Table S2.

Seventeen patients (26%) experienced lupus nephritis flares requiring hospitalization, repeat biopsy, and/or escalation of immunosuppressive therapy. Flares were managed by treating rheumatologists or nephrologists as per their clinical discretion. Table 2 shows the rate of ESRD and mortality among different LN classes.

## 4 Discussion

In this single center retrospective cohort, we show that response to treatment can depend on a multitude of factors and vary over the course of time in LN.

Women of childbearing age were predominant in our study population consistent with the demographic of LN patients.

Studies have shown that anti-dsDNA antibodies were positive more frequently in patients with proliferative than mixed proliferative LN or pure class V LN (12, 13). However, our study did not show this difference.

Eighteen patients underwent kidney biopsy for AKI, whereas many of the patients underwent biopsy for proteinuria or abnormal urine sediment. The incidence of AKI was similar in patients with proliferative LN (i.e., III, IV or overlap disease) and class V LN alone. Our study did not show statistical difference in AKI recovery between the different LN groups.

Nine patients did not recover from AKI out of which 4 progressed to ESRD. In all 4 patients' activity scores were 16 or higher, highlighting the importance of activity scores as a predictor. In keeping with other studies, high activity and chronicity scores are independent predictors of ESRD progression (14).

For Class I and II LN patients, CR and PR rates slightly reduce at 12 months. Lack of adherence, small sample size and attrition bias could be the confounding factor. For proliferative LN at 6 months, 11/39 (28.2%) patients had PR and 9/39 (23.1%) had CR. At 12 months the PR was 12.5% but CR improved to 37.5%, highlighting the importance of prolonged immunosuppression in control of the disease.

Coming to pure Class V LN, CR drops to 6.7% at 12 months. The patients in Class V had a mean chronicity score of 4 as compared to 2.2 for proliferative LN. This later translates into the highest percentage of ESRD patients in this group. Progressive CKD and ESRD are the most important predictors of mortality in LN. In our study cohort, 17% of patients (11/66) progressed to ESRD during the study period. We observed a clear separation in ESRD risk by LN class, with increasing rates of progression from Classes I and II through Class V.

There was a statistically significant difference between the classes for response rates (*p* = 0.04) at 12 months. At 12 months only 1 of the 11 patients that progressed to ESRD had met criteria for either partial or complete response. This mirrors the results seen in other studies, where proteinuria and eGFR alongside achieving renal response at 1 year were the strongest predictive markers for progression to ESRD (15, 16). In our study 9 patients died out of which 4 had ESRD. This underlines the strong correlation between renal survival and patient survival.

Presence of AKI and proteinuria, especially if nephrotic range has been linked with poor renal outcomes in many studies (17–19). Thus, reduction in creatinine and proteinuria reflects early response to therapy and is associated with a more favorable long-term prognosis (16, 18).

In the study population, 70% lacked secondary insurance. Disease modification is a crucial factor and studies show that in the real-world maintaining disease control over prolonged periods of time is difficult (20). The health care costs associated with active LN are substantial (21). In our population socioeconomic factors likely posed a barrier to achieving disease modification.

Flares of LN represent another important prognostic marker and indicator of therapeutic effectiveness. In our cohort, 26% of patients experienced LN flares requiring hospitalization, repeat biopsy, and/or escalation of immunosuppressive therapy. Recurrent flares suggest incomplete disease control and contribute to cumulative renal injury, further increasing the risk of worsening long-term outcomes as shown by Panagiotopoulos et al. and others (22–24).

Immunosuppression continues to be the double-edged sword for LN patients as it significantly increases the risk of infections. In this study, we included only the ones that lead to hospitalization. This likely underrepresents the true rate of infections as it captures only serious ones that warranted hospitalization. Of the induction groups, intravenous methylprednisolone had the highest rate of infections (100%). Cyclophosphamide induction and MMF induction had similar rates of infection, at 36% and 35%, respectively.

An interesting finding in our study was that 55% (5/9) patients who did not have induction immunosuppression experienced infections necessitating hospitalization. The possible causes for this include exposure to glucocorticoids and presumed T, B, and NK cell dysfunction could play a role in the predisposition to infections in SLE patients (25). Almost every single patient in this cohort received oral glucocorticoids contributing to immunosuppression that was not measured by induction therapy. Much of the study period coincided with the COVID-19 pandemic. One of the most common infections observed was viral pneumonia and 4 patients had COVID-19 infection. This also potentially affects any differences that may be observed by immunosuppression choice during this time as all patients were at risk of hospitalization due to COVID-19.

We acknowledge the retrospective observational nature of our data created many limitations. Small sample size limits the amount of subgroup analyses that can be conducted. Individual risk factors such as activity score and chronicity score and their impact on outcomes did not reach statistical significance likely due to sample size. Such analyses are not feasible in small sample sizes such as ours. Especially at longer timepoints follow-up data was limited, only 52 patients had reliable data at 2 years, and only 27 patients at 5 years which limits estimates of ESRD, flares, and overall mortality. The heterogeneity of reported data in chart review, with significant variability in start and end points, along with significant attrition at later time points makes analysis of predictors, especially regarding ESRD limited. With less than half of patients having data available at 5 years any statistical test of predictive factors is limited in its reliability and likely won't be able to accurately represent true risk of progression. This further highlights the need for ongoing research and data collection for long term outcomes in patients with LN as there is still a gap in long term prediction of progression and disease response.

Analyses for relapses, medication selection, and complications are largely descriptive due to the limited size of our population and difficulty in any analysis reaching the significance threshold. It is still important that these are reported because they highlight the ongoing challenges that face clinicians in practice, that aren't necessarily noticed in the limited and controlled follow-up of clinical trials.

Infections and kidney biopsy related complications severe enough to cause hospital admissions or emergency room visits were captured. Chart review failed to capture the encounters that took place outside our organization. Hence the total volume of treatment related adverse outcomes is underestimated.

The mean duration of SLE prior to biopsy in our study had very high IQR, and we could not reliably assess the correlation between the mean duration of disease and impact on survival or mortality. We also noticed heterogeneity in the provider management of LN patients with regards to choice of therapy, dose, and duration of induction and maintenance immunosuppression. Similar to a recent study, MMF was the mostly commonly used drug (26).

The study was conducted during a period when newer immunosuppressives such as belimumab and voclosporin had only recently been approved. Provider preference, as well as insurance approval were barriers to initiating newer medications.

Management of LN patients continues to pose a significant challenge despite the availability of multiple immunosuppressants. Sustained disease control, which is often dependent upon individualized treatment plans, patient adherence, socioeconomic factors, infections play a key role in preserving renal function.

Delays in biopsy and treatment increase the risk of progression and renal failure as supported in similar studies (18). In our data the median duration of lupus prior to biopsy was 12 months, hence we feel there is a need for increased awareness about LN between primary care physicians as well as specialists.

SLE and LN patients are often co-managed by multiple specialists. Despite guidelines from different societies, there is an urgent need especially for collaboration between rheumatologists and nephrologists for consistent management of these patients. Institution-dependent protocols for referrals, laboratory monitoring, and even therapeutic choices may help overcome inconsistency.

Our data shows that despite initiation of immunosuppression, response is slow, and outcomes variable. Several studies have noted that early biopsy and prompt immunosuppression are crucial in providing the most benefit to patients. LN is a complex disease, and interdisciplinary work between specialties is vital, though also contributes to the heterogeneity in management. There are likely multiple factors at play including delayed diagnosis and treatment, patient adherence, and socioeconomic interplay that contribute to an uncertain picture of outcomes and progression. More work is needed to further quantify the time to full renal response, risk of progression, and targetable prognostic indicators for patients with LN.

## Data Availability

The raw data supporting the conclusions of this article will be made available by the authors, without undue reservation.
