# Supplementary material for: Beyond the trial: a single-center retrospective review of real-world lupus nephritis management and outcomes
Source: Front Med (Lausanne). 2026 Jul 21;13:1868000. doi: 10.3389/fmed.2026.1868000 (PMC13434844; doi:10.3389/fmed.2026.1868000)
Supplement: Supplementary file 1 [file Table_1.docx]

***Supplementary Table S1. All Disease/Treatment Related Complications Noted in EMR.***

| Complication |  | N |
| --- | --- | --- |
| Bacterial Pneumonia |  | 6 |
| UTI |  | 4 |
| SARS-CoV-2 |  | 4 |
| HSV Infection |  | 4 |
| Retroperitoneal Hematoma |  | 4 |
| Acute Otitis Media |  | 2 |
| HPV Infection |  | 2 |
| Influenza |  | 2 |
| E. Coli Bacteremia |  | 2 |
| MRSA Bacteremia |  | 2 |
| Bacteremia |  | 2 |
| Viral Pneumonia |  | 2 |
| Osteomyelitis |  | 2 |
| MSSA Endocarditis |  | 2 |
| Hair Loss |  | 2 |
| Oral Ulcers |  | 2 |
| Thrush |  | 1 |
| Varicella Zoster |  | 1 |
| Candida Esophagitis |  | 1 |
| MSSA Bacteremia |  | 1 |
| Klebsiella Bacteremia |  | 1 |
| Pseudomonas Bacteremia |  | 1 |
| Enterobacter Bacteremia |  | 1 |
| Stenotrophomonas Infection |  | 1 |
| Renal Abscess |  | 1 |
| Retroperitoneal Abscess |  | 1 |
| Pleuritis |  | 1 |
| Aspergillosis |  | 1 |
| Blastomycosis |  | 1 |
| Cerebritis |  | 1 |
| Serositis |  | 1 |
| Norovirus |  | 1 |
| Cerebral Sinus Thrombosis |  | 1 |
| Burkitt Lymphoma |  | 1 |
| Craniopharyngioma |  | 1 |
| Osteoporosis |  | 1 |
| Avascular Necrosis |  | 1 |
| Anemia |  | 1 |
| Hypertensive Urgency |  | 1 |
| Cryoglobulinemia |  | 1 |
| Toe Gangrene |  | 1 |
| Perinephric Bleed |  | 1 |
