# Supplementary material for: Beyond the trial: a single-center retrospective review of real-world lupus nephritis management and outcomes
Source: Front Med (Lausanne). 2026 Jul 21;13:1868000. doi: 10.3389/fmed.2026.1868000 (PMC13434844; doi:10.3389/fmed.2026.1868000)
Supplement: Supplementary file 2 [file Table_2.docx]

***Supplementary Table S2. Causes of Death***

| Cause of Death |  |  |
| --- | --- | --- |
| Sepsis |  | 2 |
| Tumor Lysis Syndrome |  | 1 |
| Hypoxic Respiratory Failure |  | 1 |
| Subarachnoid Hemorrhage |  | 1 |
| Cardiogenic Shock |  | 1 |
| Unknown |  | 3 |
